# Integrative Genomics and Transcriptomics Analysis Reveals Potential Mechanisms for Favorable Prognosis of Patients with HPV-Positive Head and Neck Carcinomas

**DOI:** 10.1038/srep24927

**Published:** 2016-04-25

**Authors:** Wensheng Zhang, Andrea Edwards, Zhide Fang, Erik K. Flemington, Kun Zhang

**Affiliations:** 1 Department of Computer Science, Xavier University of Louisiana, 1 Drexel Drive, New Orleans LA 70125, USA; 2 Biostatistics Program, School of Public Health, Louisiana State University Health Sciences Center, New Orleans, LA, 70112, USA; 3 Tulane Health Sciences Center, Tulane Cancer Center, Tulane University, 1700 Tulane Ave, New Orleans, LA 70112, USA.

## Abstract

Patients with HPV-positive head neck squamous cell carcinomas (HNSCC) usually have a better prognosis than the HPV-negative cases while the underlying mechanism remains far from being well understood. We investigated this issue by an integrative analysis of clinically-annotated multi-omics HNSCC data released by the Cancer Genome Atlas. As confirmatory results, we found: (1) Co-occurrence of mutant TP53 and HPV infection was rare; (2) Regardless of HPV status, HNSCCs of wild-type TP53 implied a good survival chance for patients and had fewer genome-wide somatic mutations than those with a mutation burden on the gene. Our analysis further led to some novel observations. They included: (1) The genes involved in “DNA mismatch repair” pathway were up-regulated in HPV-positive tumors compared to normal tissue samples and HPV-negative cases, and thus constituted a strong predictive signature for the identification of HPV infection; (2) HPV infection could disrupt some regulatory miRNA-mRNA correlations operational in the HPV-negative tumors. In light of these results, we proposed a hypothesis for the favorable clinical outcomes of HPV-positive HNSCC patients. That is, the replication of HPV genome and/or its invasion into the genomes of cancer cells may enhance DNA repair mechanisms, which in turn limit the accumulation of lethal somatic mutations.

Head and neck squamous cell carcinoma (HNSCC) is the sixth leading cause of cancer death worldwide^1^. The five-year survival rate of patients with HNSCCs is about 40–50%^2^. The prevalence of p53 mutations in HNSCCs ranges from 30 to 70% according to various research reports^3^. Human papillomavirus (HPV) has emerged as a major risk factor for the development of HNSCCs, especially for the tumors initiated at oropharynx^4^. HPV induces cancer via infecting epithelial cells. The viral genome is typically integrated into the host cell genome in the way that the E2 open reading frame of the virus is disrupted, causing upregulated expression of the viral E6 and E7 onco-proteins that is normally suppressed by E2 protein^5^,^6^,^7^,^8^,^9^,^10^. E6 and E7 proteins bind, respectively, to and inactivate the tumor suppressor proteins TP53 and RB1, enabling the host cells to avoid apoptosis and to grow in an uncontrolled manner^11^,^12^,^13^. These infected cells are usually recognized by the immune system and eliminated^14^. Sometimes, however, they are not destroyed, and a persistent infection results. As the persistently infected cells continue to grow, they may develop mutations that promote even more cell growth, leading to the formation of a high-grade lesions and, ultimately, a malignant tumor^14^,^15^. HPV-positive HNSCCs are also characterized by high expression levels of p16 ^INK4A^ coded by the cancer suppressor gene CDKN2A^16^, which has the second highest mutation rate in TCGA head and neck cancer samples. A recent publication showed that HPV integrations in HNSCCs are associated with somatic alterations of key cancer genes and a specific methylation signature^17^.

Compared to patients with HPV-negative HNSCCs, those with HPV-positive HNSCCs have a good prognosis, regardless of the treatment strategies (e.g., surgery, radiotherapy, concurrent chemoradiation therapy, or induction chemotherapy plus concurrent chemoradiation)^4^,^18^. While the underlying mechanisms for this association remain unclear, some relevant hints can be extracted by scrutinizing the mutation spectra of HNSCCs. For example, the mutation of TP53 usually leads to a poor prognosis, and HPV infection is more frequently detected in the tumors without TP53 mutation^19^,^20^. This implies that the interplay between HPV and TP53 in HNSCCs is not merely limited to the inactivation of p53 protein by E6 that likely alleviate the need of mutations in tumorigenesis, but may also involve a mutual transcriptional or genetic interference and a further association with patient survival.

Previous studies demonstrated that the gene expression profiles of HPV-positive and HPV-negative HNSCCs are truly distinguishable^21^, especially in the genes playing roles in cell cycle process^22^. The difference between these two types is even more substantial than that between HPV-positive HNSCCs and HPV-positive cervical carcinomas (CESCs)^8^. This observation prompts the cancer community to regard HPV-positive HNSCC and HPV-negative HNSCC as two distinct cancer (sub) types in seeking therapy. Meanwhile, it also motivates researchers to relate the differentiated gene expression profiling to the differentiated somatic mutation spectra. Recently, Henderson *et al*.^23^ showed that APOBEC cytosine deaminase activity plays roles as a key driver of PIK3CA mutagenesis and HPV-induced malignant transformation in HNSCCs^23^. The main evidence for their finding is that APOBEC activity may cause helical domain mutations in PIK3CA, and APOBEC3B expression is elevated in HPV-positive HNSCC group^23^,^24^. Intuitively, this at most represents a part of the story of protein-mediated transcription-related carcinogenesis in HPV-positive tumors. This is because the cancer driver mutations in the subtype is not limited to those present in PIK3CA, and the observed mutation spectrum is often the consequence of DNA mutations and mismatch/aberration repairs. In this regard, further scrutinizing the subtype specific gene expression profile in HNSCCs may help elucidate the favorable survival rate of patients with HPV infected tumors.

MicroRNAs (miRNAs) comprise a highly conserved class of small RNA molecules (18–24 bp) that primarily bind to the 3′ UTR of mRNAs and either block translation or promote mRNA degradation. Global miRNA expression changes in HNSCCs compared to normal tissue samples and the difference between HPV-positive and HPV-negative tumors have been widely reported^25^,^26^. A recent study on cervical cancers shows that HPVs have oncogenic properties at least in part by reshaping the milieu of cellular miRNAs^27^. Shi *et al*.^28^ found that the glucocorticoid mediated regulation of a HPV-E6-p53-miRNA-145 pathway could modulate invasion and therapy resistance of cervical cancer cells^28^. These results suggest that the interference of HPVs to the miRNA-mRNA interactions may play a role in the mechanisms underlying various clinical outcomes of HNSCC patients.

In this study, we comprehensively analyzed clinically annotated multi-omics data generated by the Cancer Genome Atlas^29^ to elucidate the roles of HPVs in the prognosis of patients with HNSCCs. We first stratified the HNSCC samples by HPV infection and the mutation statuses of genes TP53 and CDKN2A. Then, we modeled and characterized the subtype-specific survival profiles, somatic mutation spectra, gene expression alterations and miRNA-mRNA interactions. Based on these results, we proposed a heuristic explanation for the favorable clinical outcomes of HNSCC patients with HPV infection. We further extended the analysis to predict the HPV status of HNSCC patients using expression signatures and to identify the alterations of miRNA-mRNA correlation network modules across various HNSCC subtypes.

## Results

### Cancer patient stratification

According to HPV infection (i.e. positive or negative) and the status (i.e. wild or mutant) of genes TP53 and CDKN2A, we stratified the 296 HNSCCs, accompanied by complete clinical and somatic mutation data, into five subtypes. The first subtype, namely tp53&cdkn2a-mut_HPV− (A1), contained HPV-negative tumors with a mutation burden on both TP53 and CDKN2A. Other four subtypes were similarly defined. They include tp53-mut_HPV− (A2), tp53-mut_HPV+ (B), tp53-wild_HPV− (C) and tp53-wild_HPV+ (D). As shown in Table 1, the co-occurrence of mutant TP53 and (positive) HPV infection was rare (n = 2), and a mutant CDKN2A was present only in the samples with a mutation burden on TP53. As subtype “B” contained only two samples, we didn’t further consider it in the subsequent analysis. It is worth noting that silent mutations were excluded from our analysis, implying that the tumor stratifications based on the genotypes of TP53 and CDKN2A genes could be potentially related to the status, i.e. normal or disrupted (altered), of p53 and p16 (or p14^ARF^) proteins.

### Subtype-specific survival profiles and somatic mutation spectra

**Figure 1:**
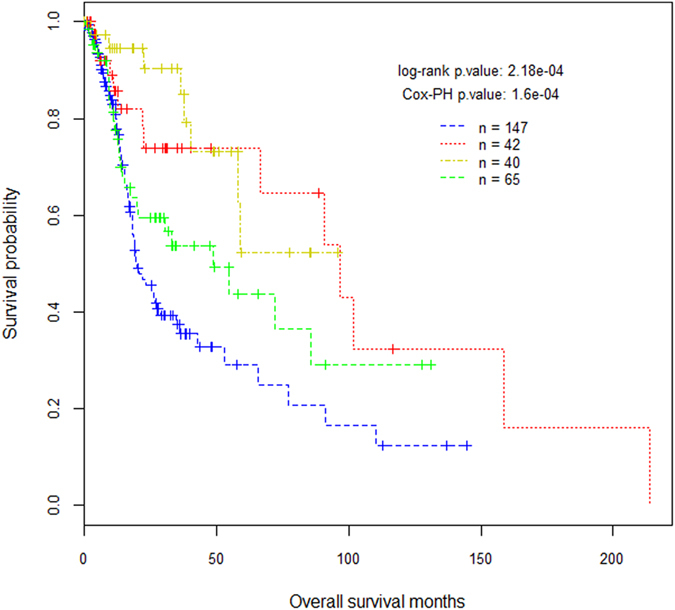
Kaplan–Meier (K-M) survival curves for patient groups defined by the proposed tumor stratification. Green (subtype A1, n = 65): tp53&cdkn2a-mut_HPV−. Blue (subtype A2, n = 147): tp53-mut_HPV−. Red (subtype C, n = 42): tp53-wild_HPV−. Yellow (subtype D, n = 40): tp53-wild_HPV+.

Figure 1 depicts the Kaplan–Meier (K-M) survival curves for the patient groups defined by the tumor stratification. Log-rank test and Cox-PH regression analysis demonstrate that, as a whole, the association between the patient survival and cancer subtypes is statistically significant before and after the initial diagnosis ages are corrected (p < 0.001). This association is primarily due to the difference between the aggregate of subtypes A1 and A2 and the aggregate of subtypes C and D. This result implies that, regardless of the HPV infection status, the patients with wild-type TP53 have better prognosis than the patients with a mutation burden on the gene. The impact of HPV infection on patient survival is demonstrated by the comparison of the K–M curves of subtypes C and D. That is, compared to subtype C, subtype D has a higher 3-year survival rate but its survival advantage is not maintained in that all patients in subtype D were deceased by year 8 while the 8-year survival rate of subtype C is over 0.5. The “double mutant” subtype A1 has a better prognosis than the “single mutant” subtype A2. Their survival curves begin to diverge at the 18 month time point and the difference is marginally significant (p < 0.07). To the best of our knowledge, this is the first work studying the relationship between CDKN2A mutation and the survival of HNSCC patients. The potential scientific merit of this observation is its biological implication (see Discussion section) rather than the prognostic value. This is because the effect of TP53 status is so drastic that it completely overrides the status of CDKN2A.

**Figure 2:**
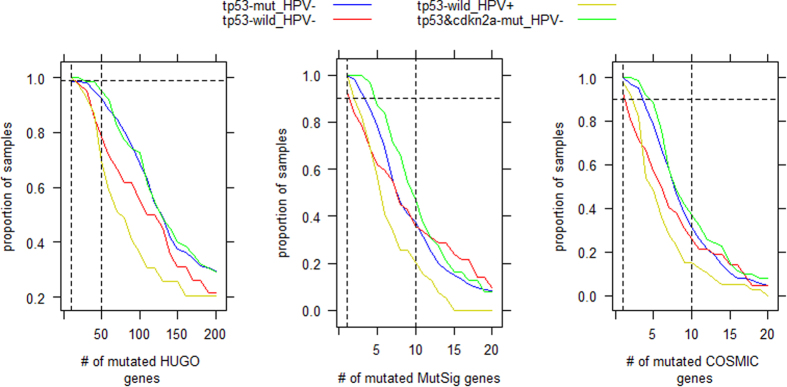
Cumulative distributions of somatic mutations present in HNSCC samples. For each gene catalogue (i.e. HUGO, MutSig or COSMIC), the sample proportion (y) corresponding to a specific mutation burden (x) is calculated by dividing the number of samples with mutation burden ≥x by the total number of samples. A specific mutation burden (x) is quantified by the number of mutated genes.

We analyzed the subtype-specific somatic mutation spectra by fitting the empirical cumulative distributions of mutations present on cancer samples. The plots displayed in Fig. 2 are based on three gene sets (catalogues). The first includes all the HUGO genes whose official symbols have been approved by Human Genome Organization. The second contains 435 “cancer driver” genes identified by a pan-Cancer project using the MutSig software^30^,^31^. The third consists of 506 cancer genes collected in the COSMIC (Catalogue of Somatic Mutations in Cancer) database^32^. We found that, similar to the case of patient survival, the major stratification factor for the number of mutations present in a tumor was the genotype of TP53 in cancer cells. That is, the samples with wild-type TP53, especially those infected by HPVs (e.g. subtype D), have fewer somatic mutations than the samples with a mutation burden on TP53 (e.g. subtypes A1 and A2). While over 30% of samples in subtype C are enriched with mutations on the MutSig cancer diver genes and COSMIC cancer genes, its mutation spectrum in the HUGO genes is similar to that of subtype D as ~20% of the samples have only 10–50 mutations. Most (~ 90%) of HPV-positive tumors have at least one mutation on the cancer genes, indicating that the progression of the cancer initiated by virus infection is driven by somatic mutations, which is similar to the cancer initialized by other carcinogens. By a set of Mann–Whitney tests, we found that the differences in mutation burden are significant (p < 0.01) between subtype D and subtype A1 (A2) with respect to all the three gene catalogues.

### Subtype-specific gene expression alterations

To investigate the tumor subtype-specific gene expression alterations in HNSCCs, we performed seven Mann–Whitney tests (or comparisons) on each gene, using the information of the “expression set” in Table 1. Specifically, tests “CTR-A1N”, “CTR-A2N”, “CTR-CN” and “CTR-DN” compared the four major subtypes (i.e. A1, A2, C and D) with normal tissue samples, respectively. Test “CTR-CD” compared subtype C to subtype D to identify the genes whose expression is impacted by HPV infection. Test “CTR-A2C” compared subtype A2 to subtype C to pinpoint the genes whose expression is associated with the genotypes of TP53. Test “CTR-A1A2” compared subtype A1 to subtype A2 to identify the genes whose expression is associated with the genotypes of CDKN2A. We didn’t further analyze the results from the last two comparisons because (1) the 50 significant genes identified from test “CTR-A2C” lack functional similarity; and (2) for test “CTR-A1A2”, in which only one gene satisfies the adopted significance criterion, i.e. the expression of CDKN2B is up-regulated in subtype A1.

For each of the other five comparisons, we performed further analyses by the following procedures. We first scanned 16000 genes that have an expression (RPKM > 2) in at least half of the tumor and normal tissue samples, obtaining the p-values and fold changes (FC) for the between-group differences. Then, we adjusted the p-values by the Benjamini-Hochberg (BH) method, and identified the differentially expressed genes by the criteria of adj.p < 0.01 and FC > t (t was set to be 2.0 for comparing tumors and normal tissue samples and 1.5 for comparing two tumor subtypes). In this way, we generated five subsets of significant genes with the sizes between 2609 and 3056 (Supplementary Table S1). Finally, we performed functional enrichment analysis on each gene subset using the DAVID tool^33^.

**Figure 3:**
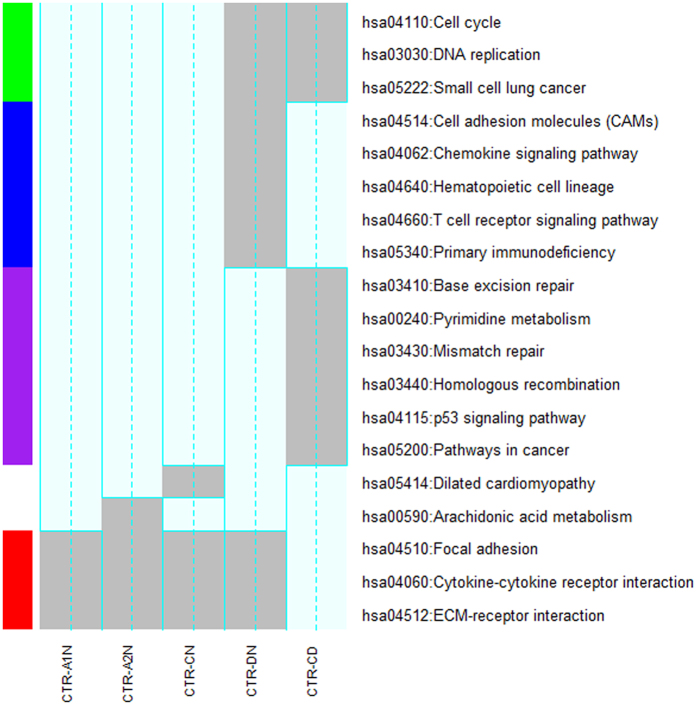
KEGG pathways over-represented by the significant genes identified in five comparisons. In this figure, an over-representation relationship (adj.p < 0.01) is highlighted in grey. The pathway clusters are determined by a hierarchical cluster analysis (Manhattan distance and Ward method) with a 0/1 matrix (i.e. M) as the input. In the matrix, rows and columns represent pathways and comparisons, respectively. When the *i*^*th*^ pathway is over represented (adj.p < 0.01) by the significant genes identified from the *j*^*th*^ comparison, the element *m*_*ij*_ of M is 1. Otherwise, it is 0.

We organized the 17 significant KEGG pathways^34^, which are over-represented (BH adj.p < 0.01) by at least one of the five gene subsets, into four pathway clusters (Fig. 3). Cluster-1 (red) includes “Focal adhesion”, “Cytokine-cytokine receptor interaction” and “ECM-receptor interaction”. They are common to all the comparisons between normal tissues and the four tumor subtypes, outlining the functional implications of the gene expression alterations in head and neck cancer cells. Cluster-2 (purple) includes “tp53 signaling pathways” and two DNA processing mechanisms, namely “Mismatch repair” and “Base excision repair”. They are unique to the comparison between HPV-positive and HPV-negative tumors and are our primary focus for further study in the next paragraph. Cluster-3 (blue) consists of “Cell adhesion molecules” and four immunity-relevant pathways such as “Primary immunodeficiency”, which characterize the virus infected tumors intuitively. Cluster-4 (green), shared by tests “CTR-DN” and “CRT-CD”, demonstrates the interference of HPV infection in cell cycle (a cancer hallmark) and DNA replication (directly related to the duplication of virus). As to this cluster, a major remaining question is why the expression alterations of cell cycle genes, as a cancer signature, were observed only in HPV-positive tumors.

**Figure 4:**
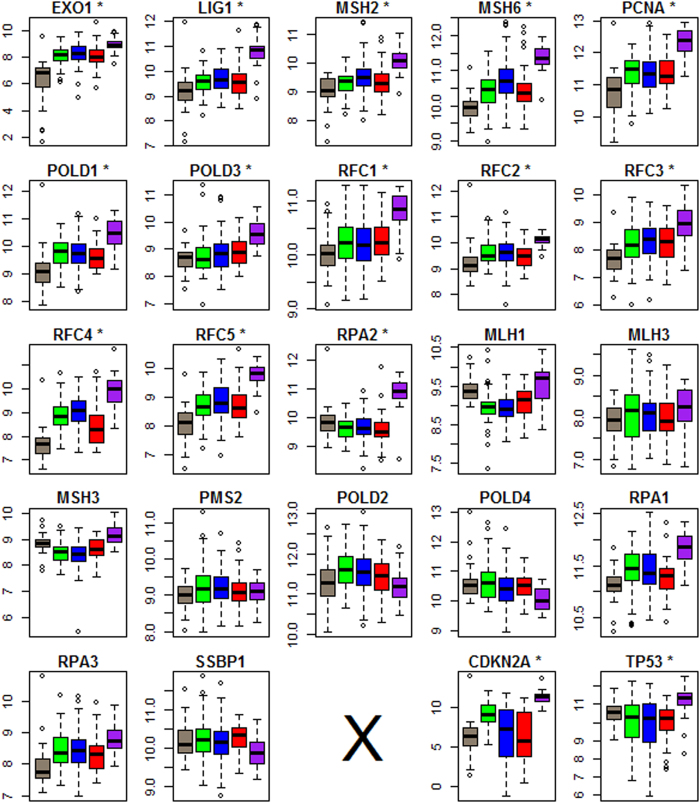
Cancer subtype-specific expression profiles of the genes in MMR pathway. Bisque: Normal tissue. Green: tp53&cdkn2a-mut_HPV−. Blue: tp53-mut_HPV−. Red: tp53-wild_HPV−. Purple: tp53-wild_HPV+. The expression profiles of CDKN2A and TP53 genes are depicted in the last two plots as a reference. The 13 significant genes in the comparison of “CTR-CD” (purple vs red) are marked with stars. Of them, the genes in subsets of (MSH2, MSH6, PCNA, RFC1-5), (EXOL1), (RPA2, POLD1, POLD3) and (LIG1) are involved in mismatch recognition, the excision of mismatched DNA, and DNA re-synthesis and ligation, respectively (http://www.genome.jp/kegg-bin/show_pathway?ko03430).

**Figure 5:**
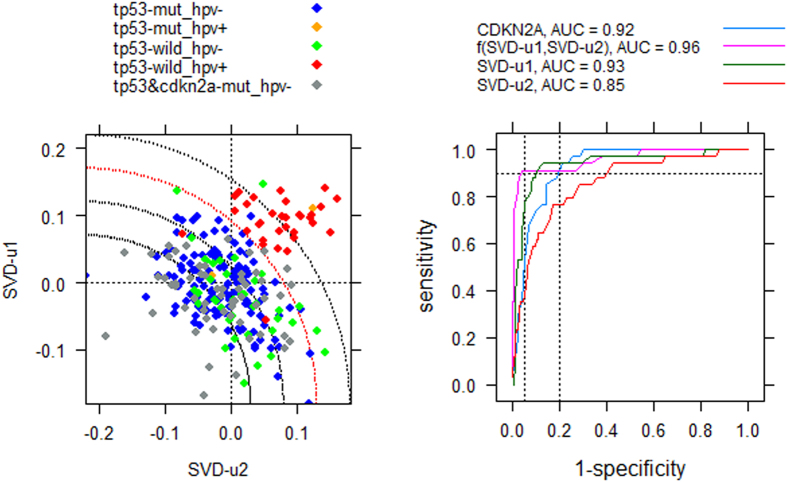
Evaluation of the expression profile of MMR genes as a prognostic signature for HPV-positive HNSCCs. Left: Illustration of the proposed SVD-based classification algorithm. SVD-u1 and SVD-u2 represent the first and second left vectors of the Singular Value Decomposition of the transpose of the row-centered expression matrix of 22 MMR genes. The score *w*_*i*_ = *f* (*u*_1*i*_, *u*_2*i*_) for the *i*^*th*^ tumor represents the distance from the corresponding data point to the center of the quarter circle. The coordinates of the center are determined by the minimums of SVD-u1 and SVD-u2. Right: Demonstration of the predictive strength of the score *w*_*i*_ as an independent predictive variable, compared with the other individual predictors, including expression level of CDKN2A, SVD-u1 or SVD-u2.

We further closely examined the tumor subtype-specific expression profiles of member genes involved in the three pathways in Cluster-2. The scrutiny demonstrated that DNA mismatch repair (MMR)^35^ had a clear relationship with the HPV status of cancer cells. Among the 23 genes annotated to MMR, RPA4 was hardly expressed in the analyzed samples and therefore was excluded from the analysis hereafter. We treated the remaining 22 genes as the “operational” MMR genes in the normal and cancer cells of head and neck tissues, and depicted their tumor subtype-specific boxplots and Mann–Whitney test results in Fig. 4. For reference purposes, we also displayed the corresponding results for CDKN2A and TP53 in the same figure. We found that over 50% of these MMR genes had a consistent transcription pattern with the expression levels in the following order, HPV-positive tumors >HPV-negative tumors >normal tissue. In particular, the transcriptionally altered genes were involved in all major steps of MMR, namely mismatch recognition, the excision of mismatched DNA, and DNA re-synthesis and ligation (See the legend of Fig. 4 for details). Based on these observations, we provided an explanation for the relationship between the subtype-specific survival profiles and the mutation spectra in the Discussion section. To study whether the 22 operational MMR genes can constitute a strong signature for the identification of HPV infection, we performed a Singular Value Decomposition (SVD) on the transpose of the row-centralized expression matrix of these genes for the 296 HNSCCs. By integrating the first and second left SVD vectors (**u_1_** and **u_2_**), we calculated a score vector ***w***, with *w*_*i*_ = *f* (*u*_1*i*_, *u*_2*i*_) being the value for the *i*^*th*^ element (sample), to separate the HPV-positive tumors from the other types (See Methods section). Using the receiver operating characteristic (ROC) analysis, we compared the predictive strength of the derived feature with that of **u_1_, u_2_** or the expression of CDKN2A, a well-known biomarker for HPV infection. In the implementation, sensitivities and specificities were tabulated at the different possible thresholds of a diagnostic test. As shown in Fig. 5, the proposed method significantly outperformed the use of **u_1_, u_2_** or CDKN2A expression alone in terms of AUC (Area Under the Curve). In particular, we achieved a classification result with both sensitivity and specificity over 0.92.

#### Validation of MMR prognostic signature

To validate the identified MMR transcriptomic signature, we performed a hierarchical clustering analysis on the GEO microarray dataset GSE3292^21^. As shown in Supplementary Figure S1, the 36 HNSCCs could be largely partitioned into two clusters and a scalar. The eight HPV-positive tumors were exclusively grouped together. The pattern of enhanced expression of MMR genes in HPV-positive tumors was clear.

### Subtype-specific miRNA-mRNA interactions

In inferring the subtype-specific miRNA-mRNA interactions, we focused on 131 miRNAs and 4875 mRNAs that show significant transcription alterations (adj.p < 0.01 and FC > 2) in at least one tumor subtype compared to the normal tissue. The transcriptional correlations (connections) between miRNAs and mRNAs were calculated by the Pearson coefficient and the significance levels were evaluated by a t-test. Based on the correlations, miRNA:mRNA interaction modules were identified by the method presented in the Material and Methods section.

**Figure 6:**
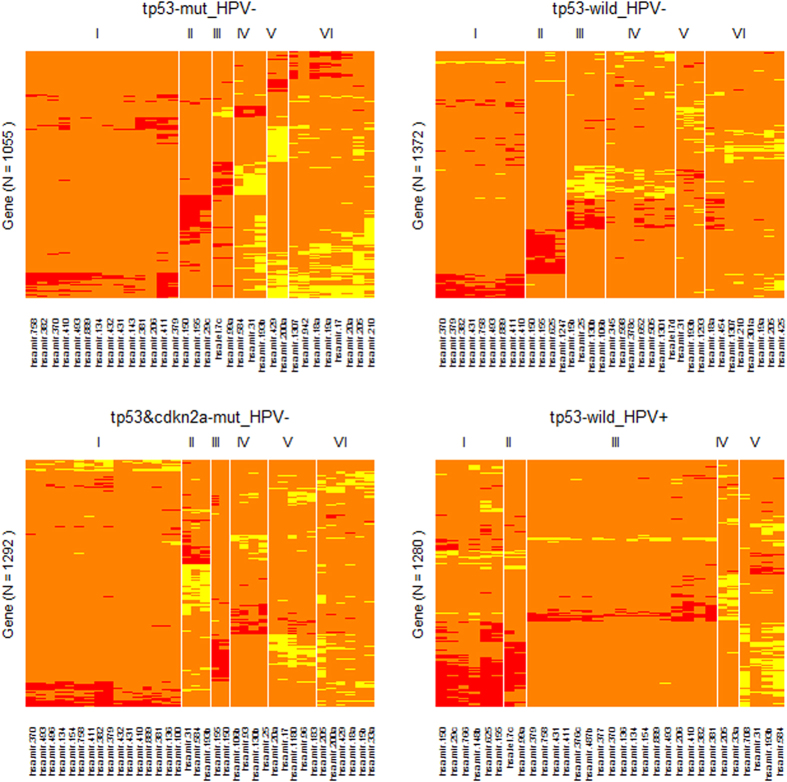
Identifying (tumor) subtype-specific miRNA-mRNA correlation-network modules by the hierarchical clustering algorithm. Red: the top positive correlations. Yellow: the top negative correlations. Orange: pseudo or unconsidered correlations. For the sake of clarification, the heatmap for each subtype was further refined by removing the columns corresponding to the miRNAs not involved in the identified modules.

As summarized in Fig. 6 and Supplementary Table S2, we identified 5 or 6 miRNA-mRNA module pairs (MPs) for each tumor subtype. Each MP included one positive-connection module and one negative-connection module. The number of miRNAs or mRNAs (genes) in each module varied from 2 to 17 or from 0 to 322. Most of the modules contain transcription factor (TF) genes that may take roles as the mediator for the miRNA-mRNA connections. The two members (such as *tp53-mut_HPV*−*/modu-I-ne* and *tp53-mut_HPV*−*/modu-I-ps*) of a MP have the same miRNAs but different mRNAs. Two modules of distinct MPs (such as *tp53-mut_HPV*−*/modu-I-ps* and *tp53-mut_HPV*−*/modu-II-ps*) consisted of different miRNAs and varied (or partially overlapped) mRNA sets. Within a positive or negative connection module (indicated by a “*-ps*” or “*-ne*” extension in the IDs), the miRNA-mRNA correlations at the expression levels were consistently positive or negative. Regardless of the connection type, the mRNAs (or miRNAs) in each module naturally represent a co-expressed gene cluster.

In the literature, it was reported that miRNAs -150 and -155 control B and T cell differentiation^36^. Here, we note that, among the positive connection modules of each tumor subtype, the one containing these two miRNAs was most significant in that the number of the modular genes was the largest and the paired negative-connection module was empty or nearly empty. Functional enrichment analysis (Supplementary Table S3) demonstrated that multiple immunity-related GO terms and KEGG pathways were over-represented by the member genes. The HPV-positive tumors differentiated from others in that multiple miRNAs (miRNA-148b,-29c,-625, and -766), along with miRNA-150 and -155, were present in the modules of subtype D. The subset of the modular genes was largely overlapped with another positive-connection module defined by let-7c and miRNA-99a. These observations implied that miRNAs were more widely involved in immunity in HPV-positive tumors than in the tumors of other subtypes.

**Figure 7:**
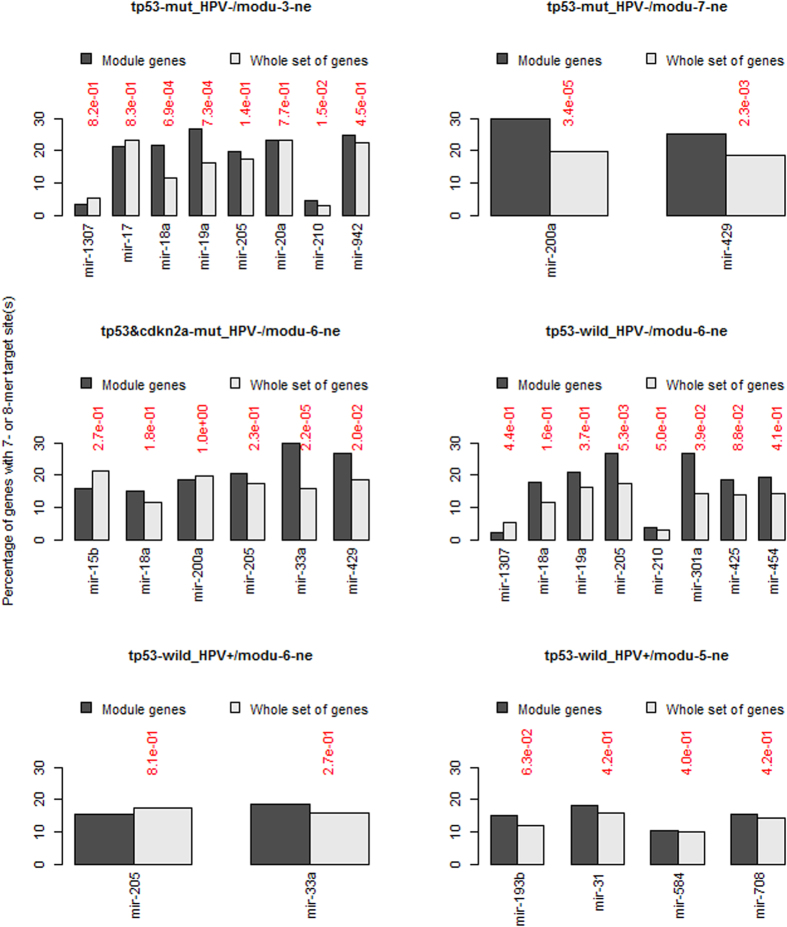
miRNA target site enrichment analysis for six miRNA-mRNA modules. The plots in the top and middle rows depict the four semi-canonical regulatory modules identified in the tumors of subtype A2, A1 or C, respectively. The plots in the bottom row depict the two major negative-connection modules identified in HPV-positive tumors (i.e. subtype D).

Among the four focused tumor subtypes, A2 had the largest sample size (N = 128), which would lead to a high statistical power in the network analysis. For this subtype, we identified two semi-canonical regulatory modules (semi-CRMs) (Fig. 7, top row), in which the 3′ UTR sequences of the involved mRNAs were enriched with the target site motifs of the modular miRNAs. Several gene ontology (GO) terms and KEGG pathways, including GO:0031012~extracellular matrix, GO:0007155~cell adhesion, hsa04512:ECM-receptor interaction and others, were over-represented by the member genes. These two semi-CRMs had approximate counterparts among the negative-connection modules of subtypes A1 and C (Fig. 7, middle row). However, neither major negative-connection modules of subtype D met the minimal requirement for a semi-CRM (Fig. 7, bottom row). These results indicated that HPV infection could disrupt some regulatory miRNA-mRNA relationships observed in the HPV-negative tumors.

## Discussion

**Figure 8:**
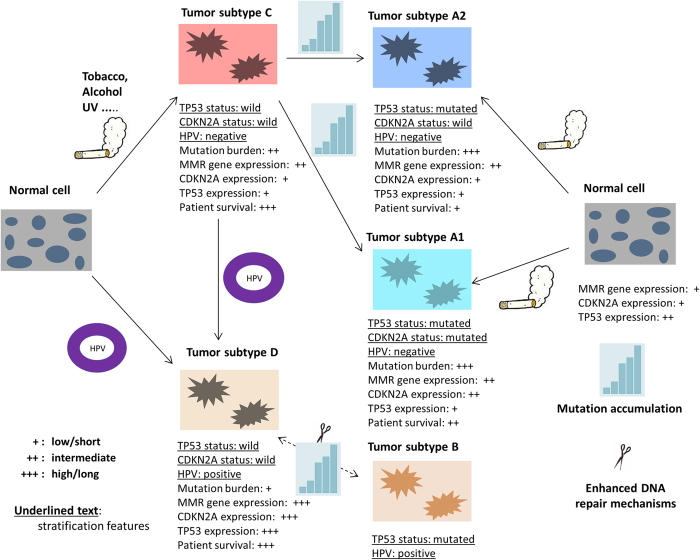
A heuristic model for the potential carcinogenesis of HNSCCs and the genetically defined progressive relationships between the different subtypes. The figure was created by the first author, Dr. Wensheng Zhang, of this paper.

Patients with HPV-positive HNSCCs have a good prognosis but the underlying mechanism remains unclear. As the major conclusion of this integrative genomics and transcriptomics analysis, we proposed a corollary hypothesis for the favorable relationship between HPV infection and patient survival. That is, the replication of HPV sequences and/or the invasion into the genomes of cancer cells may enhance the DNA repair mechanisms, which in turn limit the accumulation of lethal somatic mutations. This hypothesis is equivalent to a heuristic model describing the potential carcinogenesis of HNSCCs and the genetically defined progressive relationships between different tumor subtypes (Fig. 8). The supporting evidences include several observations (OBSs) derived from the profile of tumor subtype-specific genomic and phenotypic features.

### OBS–I

Co-occurrence of mutant TP53 and HPV infection is rare in HNSCCs. This observation confirmed the result reported by a recent TCGA publication^19^. We also notice that Smith *et al*.^37^ made a similar statement^37^. However, their claim was not sufficiently supported by the cited evidences^38^,^39^. In fact, this pattern might be masked due to the relatively weak predictive power of the utilized methods. For example, several previous studies employed the expression of p16 protein and the presence of HPV DNA (detected by a PCR-Based Mass Spectrometry System) as surrogate markers for oncogenic HPV infection^40^,^41^,^42^. However, these biomarkers (or predictive signatures) cannot guarantee high discovery specificity. As to the TCGA data focused in this study, the TP53-mut_HPV+ group contains only two samples. If the DNA presence-based technique, rather than a virus expression based method, was used to call HPV status, 22 HNSCCs would be partitioned into this subtype (“nationwidechildrens.org_clinical_patient_hnsc.txt”, a TCGA dataset downloaded on 04/25/2014). As a result, we would at most conclude that HPV infection tends to be present in wild type TP53 tumors. It is worth noting that, at present, the most robust method to detect both the presence of viral DNA and potential viral integrations, either exonic/intronic or intergenic, could be the high-pass whole-genome sequencing (WGS). TCGA measured 29 HNSCC samples with this technique. However, the method is still too expensive for a wide range of clinical applications.

### OBS-II

Regardless of HPV status, HNSCCs of wild-type TP53 imply a good survival chance for patients and have fewer genome-wide somatic mutations than those with a mutation burden on the gene. TCGA performed a more specific comparison of the mutation spectra of HPV-positive and HPV-negative tumors^19^. This phenomenon, in combination with OBS-I, indicates that the survival advantage of patients with HPV induced tumors may be due to the lack of TP53 mutation and/or low incidences of other lethal mutations. The poor association of TP53 mutation with the clinical outcome of patients has been widely reported^20^. However, this issue is complicated by the fact that a CDKN2A mutation is only observed in mutant TP53 HNSCCs and the presence improves the survival of the patients. It seems that the mutated TP53 and p16 proteins exert a very strong epistatic effect on the formation of HNSCCs but the resulting growth advantage of cancer cells is not maintained in cancer metastasis and/or the resistance to treatment therapy. In fact, the effect of a mutation on cancer cells could be positive, neutral, or negative, depending on the microenvironment and cancer progression stage^43^,^44^,^45^.

### OBS-III

The genes involved in mismatch repair (MMR) pathway were up-regulated in HPV-positive tumors compared to both HPV-negative tumors and normal tissue samples. This observation suggests a potential mechanism for the association between the mutation spectra of HNSCCs and the HPV infection status. That is, the enhanced MMR may limit the accumulation of somatic mutations as well as the occurrence of TP53 mutation in HPV-positive tumors (see next paragraph for more discussion). There are some causal factors that may be responsible for the high expression levels of MMR genes. First, the integration of virus nucleotides into the genomes of host cells could stimulate the DNA repair pathways (see Supplementary Text 1 for a note). Actually, base excision repair (BER) and nucleotide excision repair (NER) pathways, another two mechanisms for maintaining genome stability^46^, were also over-represented (adj.p < 0.025) by the significant genes (most of them are up-regulated in HPV-positive tumors) identified in the comparison of subtypes C and D. Second, replication of the HPV genome depends on the host-cell DNA replication machinery^47^ and some proteins required in the replication process play roles in MMR as well^48^. Third, the up-regulated expression of TP53 in high-stage HPV-positive tumors potentially compromises the E6/E7 (oncogenetic viral proteins) induced p53 degradation, maintaining the level of the cancer suppressor protein to activate the transcription of downstream DNA repair genes. This is unlike the situation in most HPV-negative tumors (e.g. subtypes A1 and A2) whose mutated p53 protein could lose the normal functions. This perception is supported by the result of an additional analysis of the proteomic data recently published by TCGA, that is, the HPV-positive tumors had lower TP53-R-V levels compared to HPV-negative samples but the difference was not significant (p > 0.05).

In the biological inference presented above, the hypothesis that the enhanced expression of MMR genes may limit the accumulation of somatic mutations in HNSCCs still warrants further validation, since we lack proteomic data to confirm whether the high expression levels of mismatch repair genes in HPV-positive samples can truly enhance the proteins coded by them. However, previous studies provided some indirect evidences for the assumption. For example, Hsieh and Yamane^49^ proposed that a hallmark of many MMR-deficient cells was instable at microsatellite regions consisting of mono- and di-nucleotide repeats^49^. Based on the model of *C. elegans*, Denver *et al*.^50^ found that, compared to the wild-type population, DNA repair-deficient lines with mutant gene(s) in MMR, BER and NER had higher genome-wide mutation rates, in both base substitutions and indels^50^. Their study also showed a hierarchy in the relative importance of the three repair pathways in maintaining genome stability: MMR over NER over BER. Criss *et al*.^51^ reported that mismatch correction modulated mutation frequency and pilus phase and antigenic variation in *Neisseria gonorrhoeae*^51^. Tomasetti *et al*. (2015) showed that the average somatic mutation rate in MLH1-normal colorectal cancers (CRCs) was 72% higher than that in MLH1-silent CRCs^52^.

The involvement of MMR proteins in HNSCCs has been studied recently. Theocharis *et al*. showed that the expression of the mismatch repair proteins was increased (55.1% in MSH2, 36.73% in MLH1) in tongue squamous cell carcinoma and was significantly associated with disease-free patients’ survival^53^. Pereira *et al*. reported that low expression of MSH2 DNA repair protein in HNSCCs was associated with poor prognosis^54^. These results are largely consistent with our observations and speculations. That is, HPV-positive tumors, as a whole, had high expression levels of MMR genes, and the patients with this type of cancer had good clinical outcomes.

A reviewer of this paper had an interesting question about the MMR signature. That is, is there an enhanced proliferative potential of HPV-positive HNSCCs, where the MMR involvement will be collateral as being the integral part of the post-replication repair? We addressed this issue by scrutinizing the genes differentially expressed between HPV-positive tumors and HPV-negative tumors. We found that, among the ~2700 significant genes (Supplementary Table S1) identified in the comparison of “CTR-CD”, 86 genes (subset-1) had been annotated to the Gene Ontology biological procession term “cell proliferation” (GO: 0008283) by Jan 29, 2016. In subset-1, only 41 genes (subset-2) were upregulated in HPV-positive samples compared to the HPV-negative counterparts. In this regard, it is hard to state that HPV-infected tumors are more proliferative than the others. Nevertheless, this possibility can’t be excluded yet. The reason is that subset-1 does contain genes MCM2 and KI676, which have been reported as proliferation markers^55^ and have no relation to DNA mismatch repair.

The model depicted in Fig. 8 was motivated by the mutation spectra of the entire HNSCC set. Similar to Weinberger *et al*.^40^, we displayed two potential paths for the generation of HPV-positive HNSCCs, namely tumorigenesis directly induced by HPV or initiated by other causal factors before virus infection. We further assumed that partial TP53 mutations could occur in the progression of established tumors. This proposition was supported by the similarity of gene expression profiles observed among the HPV-negative HNSCCs. The TP53/CDKN2A double-mutant tumors were considered as a special subtype (A1) due to its unique patient survival curve. CDKN2B, adjacent to CDKN2A in the human genome, was up-regulated in the tumors with mutant CDKN2A, and was the only differentially expressed gene in the comparison of “CTR-A1A2”.

In the Results section, we showed that the expression profiling of MMR genes can be used to distinguish HPV-positive HNSCCs from other tumors. The MMR signature was further validated by analyzing an independent microarray gene expression dataset. The strength of the identified signature using the proposed SVD method, in reference to the gene expression of CDKN2A alone, was well demonstrated by the obtained predictive sensitivity, specificity as well as AUC score. By an additional analysis on 172 TCGA HNSCC samples with both RNA-seq and proteomic data, we further found that the highest specificity achieved by the protein p16_INKa-R-C based prediction is 0.89 to retain a sensitivity of 0.9. This result is inferior to that obtained using the proposed method. For the subtype prediction of a new test sample *t, u*_1*t*_ (*u*_2*t*_), which are required for computing the discriminative score *w*_*t*_, can be obtained by adjusting its gene expression vector (i.e. subtracting the means of the training set from the original values) and then projecting the vector onto the product of the first (second) right singular vector and the reciprocal of the first (second) singular value of the training set-based SVD.

Recently, Parfenov *et al*.^17^ identified a methylation signature of 774 probes to distinguish HPV-positive HNSCCs from HPV-negative counterparts. We further evaluated this signature by replacing our MMR signature with it in re-performing the SVD and ROC analysis. The result showed that the predictive strength of this methylation signature was even inferior to that of the CDKN2A transcriptomic signature (Supplementary Figure S2).

Numerous studies have shown that aberrantly expressed miRNAs are likely to contribute to human diseases, including cancer. It has been recognized that the interference of miRNAs with tumorigenesis is quite complicated and needs to be scrutinized by the network-based systems biology approaches^56^,^57^. To our best knowledge, miRNA-mRNA network analysis has not been reported in head and neck cancer yet. In this paper, we initiated such a study, focusing on (tumor) subtype-specific miRNA-mRNA interactions. The results show that miRNAs are more widely involved in immunity in HPV-positive tumors than in the tumors of other subtypes in the sense that more miRNAs demonstrate modularized co-expression relationships with the genes playing roles in immune response in HPV-positive tumors. The regulatory network analysis also suggests that HPV infection may disrupt some regulatory miRNA-mRNA relationships observed in the HPV-negative tumors. It remains unclear if the disrupted regulation of miRNAs on mRNAs in cancer cells contributes to the desired survival of HPV-positive tumor patients by exerting deleterious impacts on cancer cells. Meanwhile, the observations inspire us to ponder whether non-coding HPV RNAs may serve as molecular decoys to sequester miRNAs from their target mRNAs or promote the degradation of cellular miRNAs. In this regard, it is worth noting that an evidence for virus-sourced cellular miRNA sponges was recently reported^58^,^59^.

## Material and Methods

### TCGA datasets

The analyzed TCGA clinical dataset, somatic mutation dataset (version 2.4), digital gene expression dataset (level 3) and miRNA expression dataset (level 3) are documented in the following archives, respectively. They are:

*nationwidechildrens.org_HNSC.bio.Level_2.0.5.0,broad.mit.edu_HNSC.IlluminaGA_DNASeq_automated.Level_2.1.4.0,unc.edu_HNSC.IlluminaHiSeq_RNASeqV2.Level_3.1.6.0,bcgsc.ca_HNSC.IlluminaHiSeq_miRNASeq.Level_3.1.13.0.*

The mutation calls in whole exome sequencing (WES) were validated by TCGA using a PCR-based method. The estimated false positive rate was ~ 5%^19^. The expression data have been normalized and summarized by TCGA using the standard method. We performed logarithm transformation of the expression levels before the statistical analysis.

We edited the clinical data by removing the tumors not covered by Tang *et al*.^8^. Three tumors, whose HPV-status is “intermediate” or progression-stage is “unavailable”, were also filtered. For the somatic mutation dataset, the DNA variants in the “RNA” and “Silent” categories were excluded from analysis.

### HPV status

The HPV status was defined according to the results obtained Tang *et al*.^8^. Specifically, the authors quantified viral mRNA (based on TCGA RNA-seq data) by computing the fraction of viral reads (FVR), represented as parts per million (ppm) of total library size. We considered a tumor to be HPV-positive if the FVR for any one strain of HPV was larger than 0.5 ppm. It is worth noting that the HPV status in the TCGA file of “nationwidechildrens.org_clinical_patient_hnsc.txt” was determined by a PCR and Sequenom Based Mass Spectrometry System^42^,^60^.

### Independent microarray data

A microarray dataset (GSE3292) was downloaded from Gene Expression Omnibus (GEO)^21^,^61^. In this dataset, the gene expression profiling of 28 HPV-negative HNSCCs and 8 HPV-positive HNSCCs were measured by Affymetrix Human Genome U133 Plus 2.0 Arrays. The authors of data employed quantile normalization and logarithm transformation to preprocess gene expression levels, which were estimated from the raw data by the perfect match algorithm. In the Series Matrix File, most genes are measured by two or multiple probe sets. Before the analysis, we combined the expression levels of different Affymetrix IDs for the same gene within an individual sample by calculating the average.

### Survival analysis

We performed the survival analysis using the statistical functions in the R package “survival”^62^. For a univariate survival analysis with the cancer subtype as the predictor, the function “*survdiff”* was employed to generate the Log-rank test p-value. The Kaplan-Meier survival curves in Fig. 1, with the censored observations being marked by a vertical tick, were obtained by the function “*survfit”*. A multivariate survival analysis, with “tumor subtype” and “age at initial diagnosis” as the predictors, was conducted using the function “*coxph*” that implements the Cox Proportional Hazards regression.

### SVD implementation

Suppose *M*_*p*×*q*_ is the transpose of the row centralized gene expression matrix for *q* genes on *p* tumors, the singular value decomposition (SVD) is a factorization in the form of *M* = *UDV*′^63^. In the decomposition, the *p* × *q* left factor matrix U has orthogonal columns, the *q* × *q* diagonal matrix D = diag(*d*_1_, … , *d*_*n*_) contains positive singular values ordered as *d*_*i*_ ≥ *d*_2_ ≥ … *d*_*n*_ ≥ 0, and *q* × *q* right factor matrix *V*′ has orthogonal rows and columns. Corresponding to the first two principal component vectors of matrix *M*, the leading left SVD vectors **u**_1_ and **u**_2_ can be used to partition the tumor samples into gene expression-related groups. In this paper, we proposed an *ad hoc* method to integrate them for the identification of HPV-positive tumors using the expression profiling of genes involved in mismatch repair pathway. We calculated a score vector ***w***, with *w*_*i*_ = *f* (*u*_1*i*_, *u*_2*i*_) being the value for the *i*^*th*^ element (sample). The sample was classified as HPV-positive if *w*_*i*_ was greater than the threshold. The formula for calculating *w*_*i*_ was defined as follows.


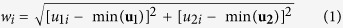


### Identification of miRNA-mRNA Modules

We employed an algorithm similar to the one used in our previous study^57^. We organized the miRNA-mRNA correlations in the form of a matrix with mRNAs in rows and miRNAs in columns. According to the signs (positive or negative), we filled the matrix with 1 or −1 for the top 2% of absolute correlations and 0 for the remaining elements. Using the matrix as the input, we generated a heatmap by applying the function “heatmap.2” in the R package “gplots”. The layout of miRNAs and mRNAs in the heatmaps was based on a two-way hierarchical clustering analysis with Manhattan distance and Ward method as the arguments. We identified the miRNA-mRNA modules by the following steps. (1) Based on the dendrogram and the miRNA-mRNA connection patterns shown on the heatmap, several modular miRNA subsets (clusters) were visually determined; (2) For each of the miRNA subsets, the positive or negative connections with mRNAs were collected into several 2-column topology matrices, respectively; and (3) A miRNA-mRNA module pair was identified from the outputs of step (2) after dropping the mRNAs with only one (positive or negative) connection.

### Target site enrichment test

Using a lab-owned R program, we identified the 7-mer and 8-mer miRNA target site motifs on the 3′ UTR sequences of the genes measured in the employed data. The binary miRNA-mRNA sequence affinity matrix (A) was then generated in a way such that an element (A_ij_) of value 1 indicated the existence of target site motif(s) for the j^th^ miRNA in the 3′ UTR sequence of the i^th^ mRNA. For a miRNA, the statistical significance of the target site enrichment level in the list of the correlated modular genes was measured by the Fisher’s exact test in reference to the level of the entire gene set.

## Additional Information

**How to cite this article**: Zhang, W. *et al*. Integrative Genomics and Transcriptomics Analysis Reveals Potential Mechanisms for Favorable Prognosis of Patients with HPV-Positive Head and Neck Carcinomas. *Sci. Rep.* **6**, 24927; doi: 10.1038/srep24927 (2016).

## Supplementary Material

Supplementary Information

Supplementary Table S1

Supplementary Table S2

Supplementary Table S3

## Figures and Tables

**Table 1:** Sample profiles and stratified subtypes.

Subtype name	Subtype ID	Mutation set size	Expression set size^a^
normal tissue	–	–	44
tp53&cdkn2a-mut_HPV−	A1	65	57
tp53-mut_HPV−	A2	147	128
tp53-mut_HPV+	B	2	2
tp53-wild_HPV−	C	42	39
tp53-wild_HPV+	D	40	32

^a^In the dataset, all normal tissue samples have mRNA-seq and mRNA-seq information, and tumor samples have somatic mutation, mRNA-seq and miRNA-seq information. In our analysis, the mutation set (N = 296) contains 275 HNSCCs that were also involved in the TCGA study (N = 279) (2015)^19^. Among these 275 common samples, only one has different HPV-status between our analysis and the TCGA study. In determining the final HPV-status of a HNSCC, TCGA considered the concordance between the RNA-seq data and other molecular and sequence information, including WGS data.
