# Supplementary material for: Integrative Genomics and Transcriptomics Analysis Reveals Potential Mechanisms for Favorable Prognosis of Patients with HPV-Positive Head and Neck Carcinomas
Source: Sci Rep. 2016 Apr 25;6:24927. doi: 10.1038/srep24927 (PMC4842993; doi:10.1038/srep24927)
Supplement: Supplementary Information [file srep24927-s1.doc]

**Supplementary Files of**

**Manuscript Title:** “Integrative Genomics and Transcriptomics Analysis Reveals Potential Mechanisms for Favorable Prognosis of Patients with HPV-Positive Head and Neck Carcinomas”,

**Authors:** Wensheng Zhang, Andrea Edwards, Zhide Fang, Erik K. Flemington, Kun Zhang

**Supplementary Table S1. Significant genes identified in between-subtype comparisons (five worksheets are included).**

**Supplementary Table S2. Summary of subtype-specific miRNA-mRNA correlation network modules (four worksheets are included).**

**Supplementary Table S3. Summary of functional enrichment analysis of genes involved in the identified miRNA-mRNA correlation modules.** The BH adjusted p-values are listed. NA indicates that either adj.p is greater than 0.01 or no gene in the module has been annotated to the GO terms (or the KEGG pathway).

**Supplementary Text 1** (page-2).

**Supplementary Figure S1** (page-3)**.**

**Supplementary Figure S2** (page-4).

**Supplementary Text 1**

In our study, 26 tp53-wild_HPV+ HNSCCs in the expression set overlapped with the 279 tumors addressed in the TGCA’s recent publication[^1^](#_ENREF_1). Among the 26 shared samples, 20 were identified as HPV-positive/integration tumors using high-pass (coverage) or low-pass Whole Genome Sequencing (WGS) technology[^1^](#_ENREF_1). Based on this information, we performed both the Mann Whitney test and t-test, and did not find a significant effect (p > 0.05) of viral integration on the expression of MMR genes in HPV-positive HNSCCs. However, this result could be subject to the small sample size of the integration-negative subgroup (N = 6). This issue might be further complicated by the potential existence of false integration-negative cases. Such a reasoning is also supported by the previous studies that showed the integration of HPV sequences is the typical way for the generation of oncogenetic viral E6 and E7 in host cells[^2^](#_ENREF_2) and nearly all HPV-positive HNSCCs express E7 and E6 (in truncated form)[^3^](#_ENREF_3).”

**References:**

1 TCGA. Comprehensive genomic characterization of head and neck squamous cell carcinomas. *Nature* **517**, 576-582, doi:10.1038/nature14129 (2015).

2 Romanczuk, H. & Howley, P. M. Disruption of either the E1 or the E2 regulatory gene of human papillomavirus type 16 increases viral immortalization capacity. *Proc Natl Acad Sci U S A* **89**, 3159-3163 (1992).

3 Tang, K. W., Alaei-Mahabadi, B., Samuelsson, T., Lindh, M. & Larsson, E. The landscape of viral expression and host gene fusion and adaptation in human cancer. *Nat Commun* **4**, 2513, doi:10.1038/ncomms3513 (2013).


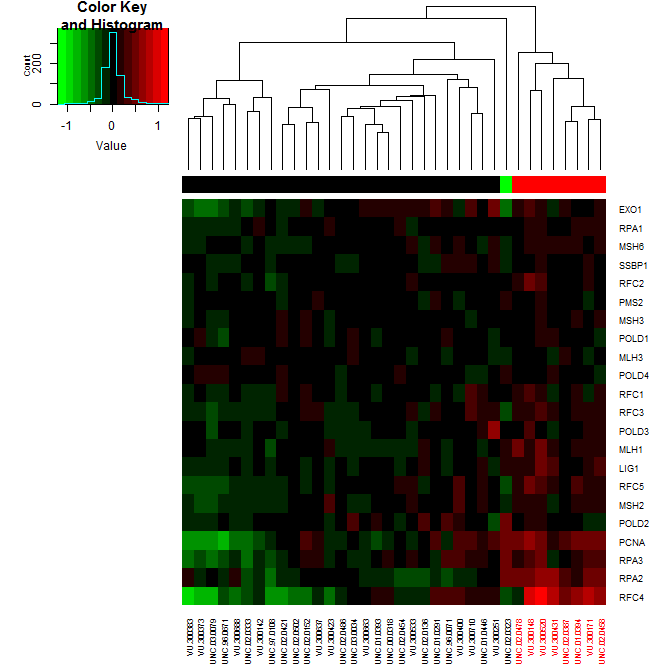


**Supplementary Figure S1.** **Validation of the expression profiling of mismatch repair (MMR) genes as a prognostic signature for HPV-positive HNSCCs**. Hierarchical clustering analysis (using “Euclidean” distance and “average” method”) was performed by the *hclust* function in the R package “stats”. The gene expression profiling was row-centralized before the analysis. The figure was generated by the *heatmap.2* function in the R package “gplot”. The column is the sample IDs, with HPV-positive tumors marked in red.


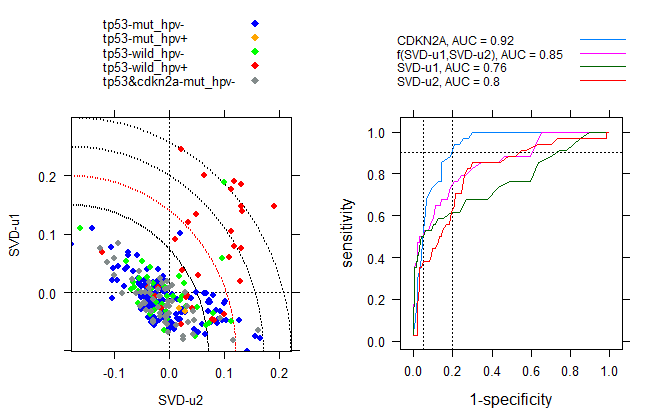


**Supplementary Figure S2. Evaluation of DNA methylation profiling as a prognostic signature for HPV-positive HNSCCs. Left:** Application of the proposed SVD-based classification algorithm. SVD-u1 and SVD-u2 represent the first and second left vectors of the Singular Value Decomposition of the transpose of the row-centered matrix of methylation percentages (beta values) of 774 predictive probes identified in (Parfenov, M. et al. 2014, PNAS, 111:15544-15549). The score *w_i_ = f (u_1i_, u_2i_ )* for the *i^th^* tumor represents the distance from the corresponding data point to the center of the quarter circle. The coordinates of the center are determined by the minimums of SVD-u1 and SVD-u2. **Right**: Demonstration of the predictive strength of the score *w_i_* as an independent predictive variable, compared with the other individual predictors, including expression level of CDKN2A, SVD-u1 or SVD-u2.
